# P-525. Severe Pediatric COVID-19 During the Predominance of the Lambda Variant at a Referral Hospital in Lima, Peru, 2020–2022

**DOI:** 10.1093/ofid/ofaf695.740

**Published:** 2026-01-11

**Authors:** Giancarlo Alvarado-Gamarra, Carlos R Celis, Katherine Alcalá-Marcos, Sara Chávez-Alvarado, Daniel Medina-Neira, Leslie C Cabrera Toribio, Claudio F Lanata, Matilde Estupiñan-Vigil

**Affiliations:** Hospital Nacional Edgardo Rebagliati Martins/ Instituto de Investigación Nutricional, Lima, Lima, Peru; Instituto de Investigación Nutricional, Lima, Lima, Peru; Instituto Nacional Cardiovascular “Carlos Alberto Peschiera Carrillo” - INCOR, Lima, Lima, Peru; Hospital Nacional Edgardo Rebagliati Martins, Lima, Lima, Peru; Hospital Nacional Edgardo Rebagliati Martins, Lima, Lima, Peru; Cincinnati Children’s Hospital Medical Center, Lima, Lima, Peru; Instituto de Investigación Nutricional, Lima, Lima, Peru; Hospital Nacional Edgardo Rebagliati Martins, Lima, Lima, Peru

## Abstract

**Background:**

Lambda (C.37) was a variant originally identified in Peru, its impact on severe COVID-19 disease in children has not been adequately studied in Andean countries. Thus, this study aims to evaluate the risk of severe COVID-19 in hospitalized children during the predominance of the Lambda variant at a referral hospital in Lima, Peru.

**Figure 1.**
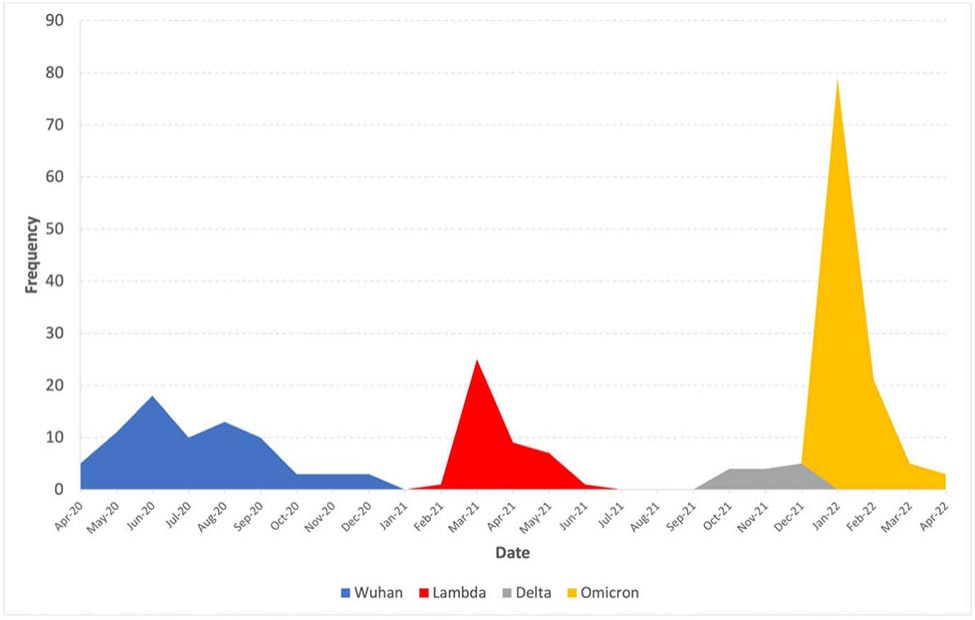
Cases of hospitalized children across periods of SARS-CoV-2 variant predominance a at Hospital Nacional Edgardo Rebagliati Martins in Lima, Peru, 2020–2022 (N=240). a. The type of SARS-CoV-2 predominance was defined as the period in which a variant was dominant (more than 70%) in Peru, according to CDC Peru and GISAID Data Science Initiative.

**Table 1.**
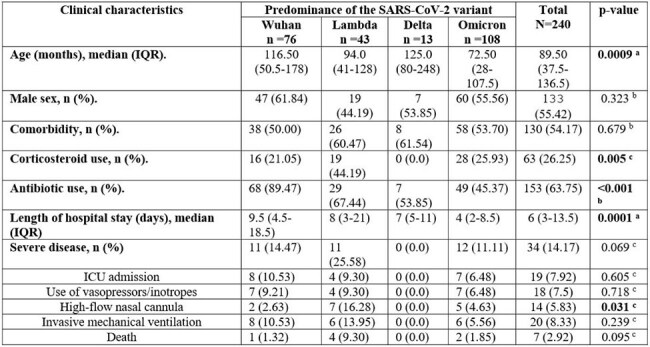
Clinical characteristics of hospitalized children with SARS-CoV-2 infection in Lima, Peru, 2020–2022. IQR: Interquartile range; ICU: Intensive care unit. a. Kruskal-Wallis test. b. Chi-square test. c. Fisher's exact t-test.

**Methods:**

Retrospective cohort of patients < 14 years hospitalized at Hospital Nacional Edgardo Rebagliati Martins, from April 2020 to April 2022. The type of SARS-CoV-2 predominance was defined as the period in which a variant was dominant (more than 70%) in Peru, according to CDC Peru and GISAID Data Science Initiative. Severe COVID-19 was defined if any of the following criteria was present: admission to the intensive care unit, use of vasopressors/inotropes, need for high-flow nasal cannula (HFNC) therapy, requirement for invasive mechanical ventilation, or death. Crude and adjusted relative risk with 95%CI were calculated using generalized linear models with a Poisson family, log link, and robust variance.

**Table 2.**
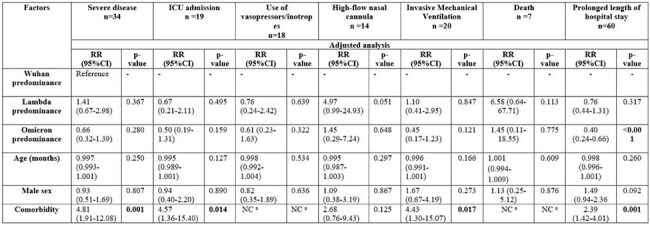
SARS-CoV-2 variants and severe COVID-19 in hospitalized children in Lima, Peru, 2020–2022 (N=240). ICU: Intensive care unit; RR: Relative risk; CI: Confidence Interval; NC: Not calculable. a. Not calculable because there is a zero value in one cell.

**Results:**

240 children were included; 18% were admitted during the period of Lambda variant predominance, median age was 89.5 months, and 54.2% with comorbidities. Severe COVID-19 occurred in 14.17%. No severe COVID-19 cases were reported during the Delta predominance, whereas 25.28% of children experienced severe disease during the Lambda predominance, with a higher use of HFNC (16.28%) compared to other variant predominance periods (p=0.031). However, the hospitalization during Lambda predominance was not associated with severe disease (RR 1.41, 95%CI:0.67–2.98; p=0.367), nor was it associated with other critical outcomes when compared to the Wuhan predominance period, adjusted for the Omicron predominance, age, male sex, and comorbidities.

**Conclusion:**

In our cohort, hospitalization during the Lambda predominance was probably not associated with greater severity of COVID-19 disease. Large multicenter studies are needed to confirm our findings.

**Disclosures:**

All Authors: No reported disclosures

